# Polymicrobial abscess following ovariectomy in a mouse

**DOI:** 10.1186/s12917-019-2125-0

**Published:** 2019-10-24

**Authors:** Victoria E. Eaton, Samuel Pettit, Andrew Elkinson, Karen L. Houseknecht, Tamara E. King, Meghan May

**Affiliations:** 1 Department of Biomedical Sciences, University of New England, 11 Hills Beach Road, Biddeford, ME 04005 USA; 2 Center of Excellences in the Neurosciences, University of New England, 11 Hills Beach Road, Biddeford, ME 04005 USA

**Keywords:** Ovariectomy, Abscess, Polymicrobial abscess, Mouse, *Streptococcus acidominimus*, *Pasteurella caecimuris*, *Gemella*, *Gemella muriseptica*

## Abstract

**Background:**

Ovariectomy is a common procedure in laboratory rodents used to create a post-menopausal state. Complications including post-surgical abscess are rarely reported, but merit consideration for the health and safety of experimental animals.

**Case presentation:**

A female C57/black6 mouse was ovariectomized as part of a cohort study. At Day 14 post-surgery, she developed a visible swelling on the right side, which 7 days later increased in size over 24 h, leading to euthanasia of the animal. Gross pathology was consistent with abscess. A core of necrotic tissue was present in the uterine horn. Abscess fluid and affected tissue were collected for Gram stain and bacteriological culture. The abscess core and fluid yielded three distinct types of bacterial colonies identified by 16S ribosomal RNA sequencing as *Streptococcus acidominimus*, *Pasteurella caecimuris*, and a novel species in the genus *Gemella*.

**Conclusions:**

This is the first report of polymicrobial abscess in a rodent as a complication of ovariectomy, and the first description of a novel *Gemella* species for which we have proposed the epithet *Gemella muriseptica*. This presentation represents a potential complication of ovariectomy in laboratory animals.

## Background

Ovariectomy (OVX) is a commonly-used model of postmenopausal age in rodent models of osteoporosis and bone fragility, impacts of menopause on dopaminergic neurons and other forms of neurodegeration, and adulthood cell differentiation [1–4].

Infection and specifically abscess is known as a potential complication of survival surgeries in laboratory mice [5]; however, information on the frequency and most common causative agents is surprisingly sparse. The most common agents of spontaneously occurring skin abscess in pet rodents are *Staphylococcus aureus*, *Streptococcus pyogenes*, and *Pasteurella pneumotropica*; however, (naturally occurring) intra-abdominal abscess is rarely discussed [6]. As prey animals, mice instinctively do not display overt signs of distress during moderate pain or sickness as a means of avoiding predation [7]. Post-surgical infection can be difficult to detect in rodents lacking overt sickness behaviors, perhaps leading to an underestimation of its rate of occurrence. Here we report a case of polymicrobial abscess following OVX in a mouse exhibiting normal behavior and no overt signs of distress.

## Case presentation

### History

A female C57/black6 mouse (Charles River Laboratories, Wilmington, MA) underwent OVX as previously described [8] as part of an 8 animal cohort. All surgical tools and silk sutures were sterilized prior to use, and the surgical field was disinfected with chlorhexidine followed by betadine. The surgical site on the mouse was sterilized with 70% ethanol followed by betadine prior to incision. All OVX procedures for the cohort proceeded unremarkably.

### Clinical findings

**Fig. 1:**
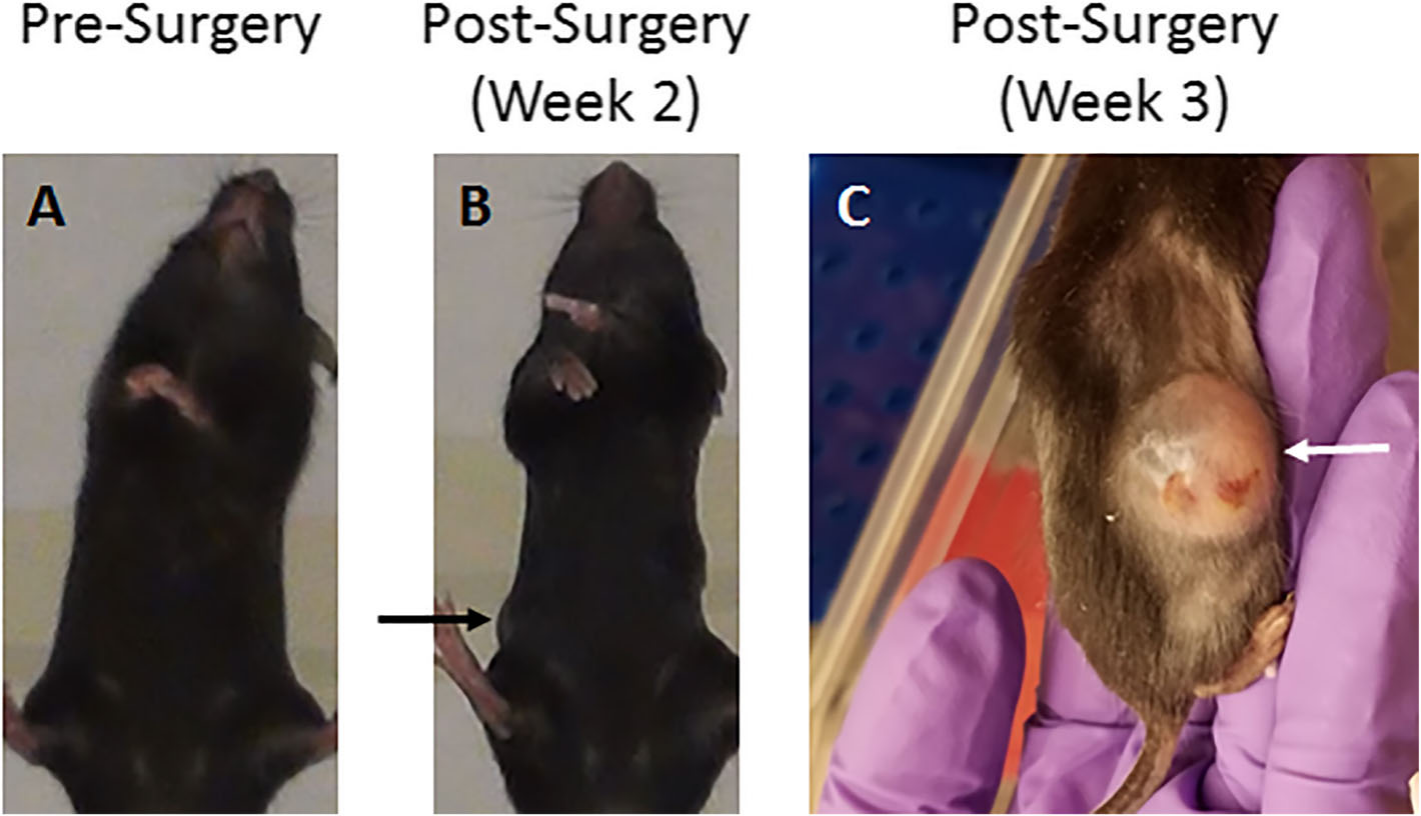
Post-Surgical Evaluation. The case study subject mouse had no apparent swelling prior to OVX (panel **a**), and slight swelling became apparent 2 weeks post-OVX (panel **b**; black arrow). The abscess increased substantially in size overnight during Week 3 (panel **c**; white arrow) leading to the immediate euthanasia of the animal

Two weeks following OVX, a slight swelling became apparent on the right side of the abdomen (Fig. 1b). Close monitoring indicated minimal growth of the swelling and no apparent impacts on behavior, weight, or food intake. No other signs of distress such as porphyrin rings around the eyes or rough hair coat or hair loss were observed by the experimenters or care staff. On days 20 to 21 post-OVX, the swelling increased substantially in size within 24 h (Fig. 1c). Though the subject was still showing no signs of distress or sickness behavior, she was euthanized because overt swelling/abscess is a humane endpoint criterion for rodents [9]. Euthanasia was carried out by pumping 100% CO_2_ into a sealed chamber at 25% chamber volume per minute, per approved IACUC protocol. No other animals in the cohort developed abscesses.

**Fig. 2:**
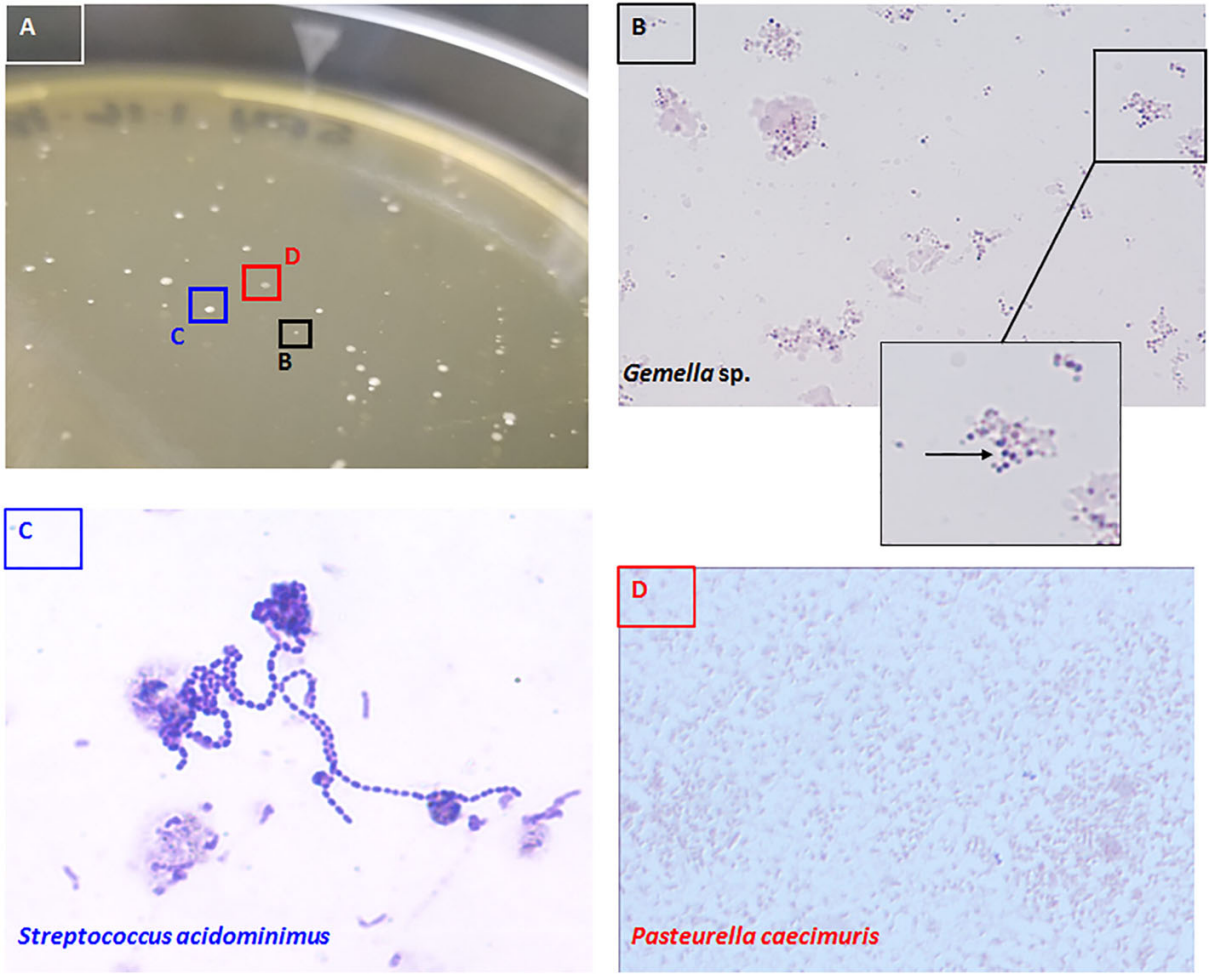
Bacteriological Identification. Three colony types were recovered on SP-4 agar (panel **a**). Each type was identified as a distinct organism, as shown by Gram stain in panels (**b**-**d**). *S. acidominimus* (**c**) and *P. caecimuris* (**d**) showed classic morphology and arrangements of their respective genera. The novel *Gemella* species (‘*G. muriseptica*’) were Gram positive diplococci (Panel **b** inset, arrow), and bacteria tended to aggregate serum proteins present in the SP-4 broth

Postmortem evaluation was consistent with post-surgical abscess. Purulent exudate was apparent into the peritoneal cavity, and a small, walled-off abscess core was located within the uterine horn. Exudate from the peritoneum and the abscess core were subjected to bacteriological culture for typical and atypical organisms using blood agar (Remel via Thermo Fisher Scientific, Waltham, MA) and SP-4 agar supplemented with 10% glucose and 15% fetal bovine serum [10]. Both specimens yielded growth of two colony types on blood agar and three on SP-4 (Fig. 2a).

### Diagnostic procedures

Colonies were propagated in axenic broth culture (SP-4 broth) for preservation, Gram stain, and molecular identification. Genomic DNA was extracted using EasyDNA reagents (Invitrogen via Fisher Scientific, Waltham, MA), and the 16S rRNA gene was amplified as previously described using the following primer sequences: 5ˈ AGAGTTTGATCCTGGCTCAG 3ˈ; 5ˈ ACGGCTACCTTGTTACGACTT 3ˈ [11]. Amplicons were subjected to Sanger sequencing using standard di-deoxy labelling methods (GeneWiz, Inc. Piscataway, NJ). Bacterial identification was made via BLAST analysis [12].

### Bacteriological findings

The first colony type could only be cultivated on SP-4 agar and consisted of Gram positive diplococci (Fig. 2b). Ribosomal RNA sequencing identified this organism as a member of the genus *Gemella*, but the highest identity (*Gemella palaticanis*, GenBank accession JN713253) was only 95%. Threshold for ribosomal identity between bacterial species is widely considered 97.5% [13]; therefore, this isolate likely represents a novel, previously undescribed species. The second colony type organisms were Gram positive cocci that formed long chains (Fig. 2c) and were identified as *Streptococcus acidominimus* (98% identity with *S. acidominimus* strain LMG 17755, GenBank accession NR_104972). The third colony type organisms were Gram negative rods (Fig. 2d) and identified as *Pasteurella caecimuris* (99% identity with *P. caecimuris* strain AA-424-CC-1, GenBank accession NR_144618).

### Gemella *Species Characterization*

**Fig. 3:**
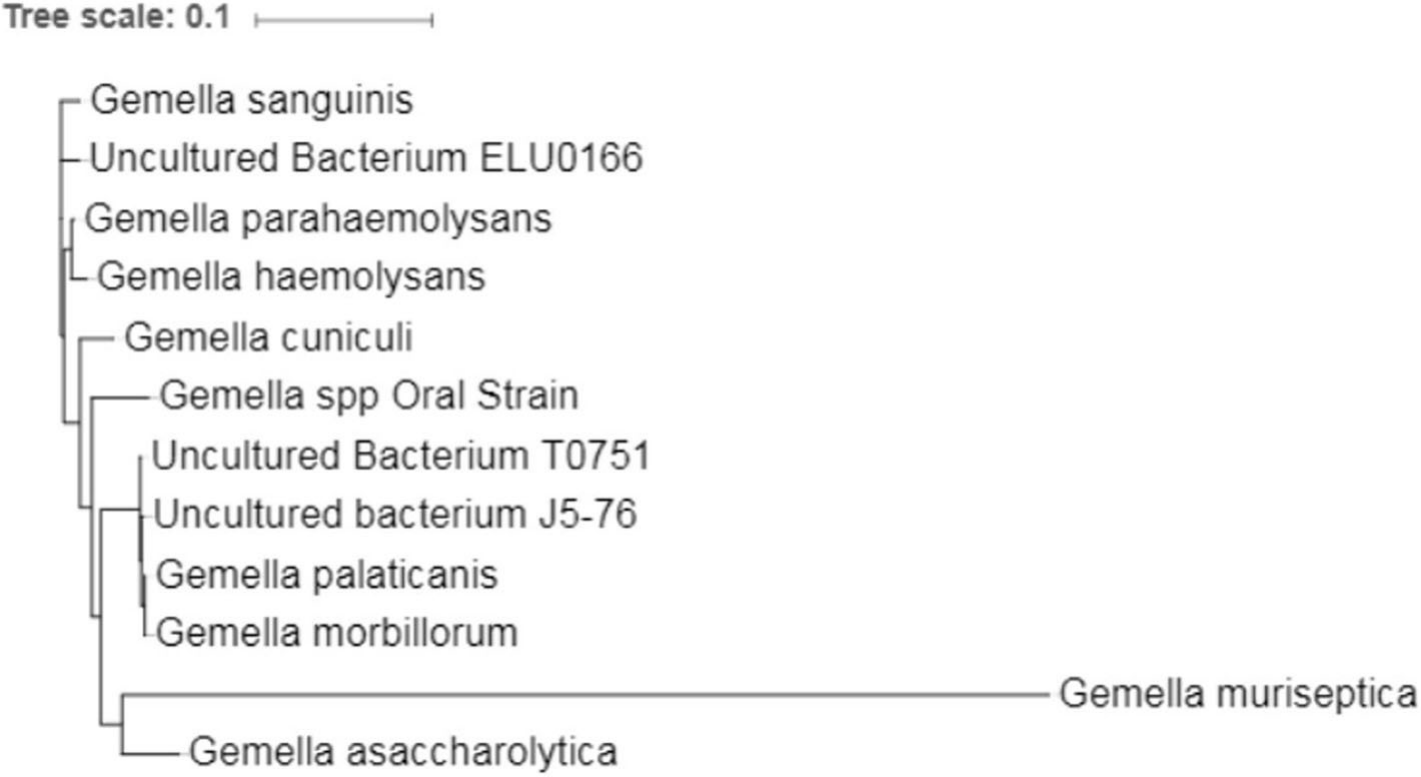
Phylogeny of *Gemella muriseptica*. An unrooted neighbor-joining tree was generated based on 16S rRNA gene sequences from several species in the genus *Gemella*, as well as uncultivated sequences obtained from mammalian metagenomic studies. *G. muriseptica* is a divergent member of the genus, and is closest branching relative is *Gemella asaccharolytica*

An unrooted, neighbor-joining tree based on 16S ribosomal RNA sequence from several *Gemella* species was generated using MEGA version 7.0 [14](Fig. 3). Input sequences were obtained from GenBank, and included those from *G. palaticanis*, *Gemella sanguinis*, *Gemella parahaemolysans*, *Gemella haemolysans*, *Gemella cuniculi*, *Gemella morbillorum*, *Gemella asaccharolytica*, *Gemella* species Oral Strain, and three uncultivated species from metagenomics studies (‘uncultivated bacterium’ ELU0166, T0751, and J5–76). Phenotypic characterizations were made using the BBL Crystal Identification reagents (Becton, Dickinson and Company, Franklin Lakes, NJ) (Gram Positive and Gram negative non-fermenter reagents), 5% sheep’s blood agar, SP-4 agar supplemented with 10% lactose, and phase-contrast microscopy. Phenotypic traits are shown in Table 1, and are consistent with the determination that this isolate represents a novel species.

**Table 1:** *Gemella muriseptica* Phenotypic Traits

Carbohydrate Utilization	Rxn	Amino Acid Degradation	Rxn	Additional Phenotypes	Rxn
Sucrose	+	Proline	–	β-glucosidase	+
Arabinose	+	Arginine	–	α-glucosidase	+
Maltose	+	Methionine	+	Cellobiosidase	–
Dextrin	+	Phenylalanine	+	β-glucoronidase	–
Mannitol	+	Valine	–	Phosphatase	+
Galactose	+	Tryptophan	–	β-galactosidase	–
N-acetylglucosamine	+	Glutamic acid	+	Urease	–
Trehalose	+	Ornithine	+	Catalase	–
Mannose	+	Lysine	–	Oxidase	–
Melibiose	+	Glycine	–	Esculin hydrolysis	–
Rhamnose	+			Hemolysis	γ
Sorbitol	+			Citrate utilization	–
Adonitol	+			N-acetylglucosaminidase	–
Inositol	+			α-arabinosidase	–
Lactose	–			Motility	–

Gemella muriseptica, *species nova*. *Gemella muriseptica* (mur.i.sepˈti.ca. L. neut; L. fem. *Muris*- pertaining to mice; *septica-* indicating infection. *Muriseptica-* from an infected mouse). Cells are small, Gram positive cocci appearing in pairs or tetrads. Catalase- and oxidase-negative. Colonies grow on sheep’s blood agar within 72 h and are non-hemolytic. Sucrose, arabinose, maltose, glycogen, mannitol, galactose, N-acetyl-glucosamine, trehalose, mannose, melibiose, rhamnose, sorbitol, adonitol, and inositol are used as carbon sources. The type strain is T3C1^T^, isolated from a murine abscess.

## Discussion and conclusions

Here we report a case of postsurgical polymicrobial abscess in a laboratory animal. The biphasic nature of disease progression suggests that a slow-growing abscess caused by one organism was superinfected by the second and third, although biphasic abscess has been reported during *G. morbillorum* monoinfection [15, 16]. Because all three bacterial isolates were cultivated from both specimens, it is not possible to determine the order of infection.

This case features the report of a novel *Gemella* species. While no members of the genus have been detected in rodents previously, metagenomic studies show that they are common inhabitants of the oral cavity and intestinal tracts of other mammals and can be detected in wastewater [17–20]. In addition, they are known to be rare causes of endogenous infection (including abscess) in humans [21–26]. This isolate is phenotypically distinct from other species, while retaining the traits required for inclusion within this genus (i.e., positive Gram stain, coccus shape, lack of motility, and absence of catalase and oxidase production) [27]. We propose the specific epithet *Gemella muriseptica*, species nova, for this isolate. This report also features the first isolate of *S. acidominimus* grown in axenic culture from a rodent. However, it is clear from previous metagenomics analyses that this organism is a common inhabitant of the murine microbiota (GenBank accession numbers FJ893080, KF658228, KJ910367), ( [28]), and that it has the potential for abscess formation in mice (GenBank accession number JF912770). Detection of *P. caecimuris* is not necessarily surprising, given its presence in the gut microbiome of healthy mice [29] and the ascription of *P. pneumotropica*, which holds some taxonomic confusion with *P. caecimuris* [30], to rodent abscess.

The relative rarity of reports describing post-surgical abscess in mice is potentially very consequential. Moderate levels of pain and distress can be notoriously difficult to detect in rodents because readily revealing injury or distress makes prey animals more vulnerable to predation [7]. While overt sickness behaviors occur with moribund animals, lack of expression of distress due to infection could lead to the potentially erroneous conclusion that post-surgical infection is extremely rare [31]. In an attempt to quantify the potentially confounding effects unapparent post-surgical infection could have on subsequent experiments, Bradfield et al. introduced bacteria following routine survival surgeries to induce post-surgical infections. Despite the absence of classic signs of distress, infection in rats significantly altered behavioral testing (open field apparatus) including exploratory and freezing behavior [32]. The volume and severity of inflammatory exudate in our case report subject indicates a systemic inflammatory response was ongoing despite an absence of distress and sickness behavior. Post-surgical abscesses within the abdomen do not necessarily cause the visible swelling that was present in our subject animal. Given that OVX is a common procedure in the in vivo study of osteoporosis and neurodegenerative diseases, conditions that would undoubtedly be affected by elevated inflammatory responses, post-surgical abscess is an important consideration to avoid confounding factors in rodent studies. While routine screening of blood for inflammatory markers of all subject animals in behavioral studies is likely impractical or cost-prohibitive, researchers employing behavioral testing may wish to consider collecting whole blood during necropsy and measuring white blood cell counts or inflammatory markers known to be elevated in the presence of abscess [32] in subjects displaying responses that substantially deviate from other members of their cohort.

## Data Availability

Ribosomal RNA sequences have been deposited into GenBank, and are available under accession numbers MK167981, MK168019, and MK168057. Axenic cultures of bacterial isolates are available upon request with appropriate documentation to receive bacterial cultures.

## Abbreviation

OVX: Ovariectomy
